# Evaluation of therapeutic potential of VB-001, a leave-on formulation, for the treatment of moderate adherent dandruff

**DOI:** 10.1186/s12895-017-0058-5

**Published:** 2017-05-03

**Authors:** Anamika Bhattacharyya, Nilu Jain, Sudhanand Prasad, Shilpi Jain, Vishal Yadav, Shamik Ghosh, Shiladitya Sengupta

**Affiliations:** 1 Vyome Biosciences Pvt. Ltd, Plot# 465, F.I.E., Patparganj Industrial Area, Delhi, 110092 India; 2 Medicine & HST, Brigham and Women’s Hospital, Harvard Medical School, Room 317, 65 Landsdowne Street, Cambridge, MA 02139 USA

**Keywords:** Scalp, Dandruff, Malassezia, Anti-fungals

## Abstract

**Background:**

Dandruff is a common scalp condition characterized by excessive scaling and itch. Aberrant colonization of the scalp by commensal *Malassezia* spp. is a major contributor in the multifactorial etiology of dandruff. Literature based understanding of *Malassezia* linked pathophysiology of dandruff allowed us to comprehend a strategy to potentiate the efficacy of a known antifungal agent used in dandruff therapy.

The aim of this study was to determine the efficacy and skin safety of VB-001 antidandruff leave-on formulation in comparison with marketed antidandruff ZPTO shampoo in patients with moderate adherent dandruff of the scalp.

**Methods:**

Healthy males or females aged ≥ 15 years and ≤ 65 with a clinical diagnosis of moderate adherent dandruff of the scalp were recruited for the study to monitor the effects of topical VB-001 versus those of marketed antidandruff ZPTO shampoo.

**Results:**

168 subjects were randomized to the treatment (VB-001, *n* = 84) and control (ZPTO shampoo, *n* = 84) groups. The efficacy of each product was evaluated by comparing proportion of subjects who have shown reduction in flaking by ASFS (adherent scalp flaking score) and pruritus by IGA (investigator global assessment) score. VB-001 imparted consistently better reduction in ASFS and enabled early reduction of pruritus in comparison to marketed ZPTO shampoo.

**Conclusion:**

VB-001, a leave-on formulation with ingredients chosen to selectively disturb the *Malassezia* niche on dandruff scalp by denying extra nutritional benefits to the microbe, provides unique advantages over existing best in class ZPTO shampoo therapy. It has the potential to emerge as an attractive novel treatment for moderate adherent dandruff.

**Trial registration:**

CTRI Registration number: CTRI/2013/01/003283. Registered on: 02/01/2013

## Background

Dandruff is a common scalp disorder characterized by flaking, often pruritic skin and affecting more than half of the human population [1]. The onset and development of dandruff is determined by multiple factors including abnormal colonization of skin by *Malassezia* spp. [2], sebum production [3] and individual predisposition [4, 5]. Sebaceous lipids on the skin surface support colonization and growth of *Malassezia* by fulfilling the latter’s obligate need for fatty acids as the microbe lacks fatty acid synthase genes [6]. To compensate, *Malassezia* secretes multiple lipases [6] to metabolize triglycerides in sebum and release free fatty acids for its use. Apart from acting as food for growth of *Malassezia*, these free fatty acids are thought to act as skin irritants and induce inflammatory responses typical of dandruff [3]. In susceptible individuals, the effect is amplified as the fungus not only resides on the skin surface, but reaches deeper due to inherent epidermal barrier defects of the individual.

Understandably, a common approach for the treatment of dandruff and its symptoms is to use anti-fungal agents in topical formulations to eradicate *Malassezia* from the scalp [7]. Few of the most commonly used and effective anti-fungal agents for dandruff are zinc pyrithione (ZPTO), selenium sulfide, ciclopirox and ketoconazole in wash-off formulations [8]. Despite high in vitro potency of these antifungal agents against *Malassezia* species, currently available wash-off antidandruff formulations are often limited in efficacy due to short contact time and thus fail to completely eliminate the fungus from skin. Interestingly, certain previous studies [9, 10] as well as our own in vitro screening assays (data not shown) have shown susceptibility of *Malassezia* spp. to certain medium chain fatty acids. In the light of this information, inclusion of medium chain fatty acids (or their derivatives) in topical formulations can be considered for treating *Malassezia*-induced skin infections.

The scope to formulate a more efficacious anti-dandruff product (compared to existing ones) devoid of growth promoting fatty acids (that act as food for the microbe) but including inhibitory fatty acids for *Malassezia* spp. was explored. The idea was to attempt changing the microenvironment of the dandruff-causing fungus on scalp through molecular replacement of sebaceous nutrients with a detrimental medium chain fatty acid derivative thus allowing overall higher anti-dandruff efficacy in the presence of a known antifungal agent. Thus, a leave-on antidandruff product VB-001 was formulated combining piroctone olamine and a derivative of a medium chain fatty acid, the combination showing enhanced in vitro fungal killing compared to a formulation containing piroctone olamine alone (data not shown).

The aim of this study was to evaluate the efficacy of VB-001 against a marketed reference antidandruff ZPTO shampoo (1%) in patients with moderate adherent dandruff. VB-001 demonstrated better clinical efficacy compared to the marketed wash-off antidandruff formulation containing 1% ZPTO.

## Methods

### Objectives

The general objective of this study was to determine the efficacy and in-use skin safety of VB-001 antidandruff leave-on formulation in comparison with marketed antidandruff ZPTO shampoo in patients with moderate adherent dandruff of the scalp. The specific objectives were to determine if outcomes in patients treated with VB-001 differed significantly from those in patients treated with antidandruff shampoo in terms of: (i) Mean adherent scalp flaking score (ASFS) after 2 weeks of daily application of VB-001 for a minimum period of 8 h per day (overnight) compared to a rinse-off antidandruff shampoo used on alternate days. (ii) Score of pruritus (Investigators' Global Assessment-IGA) 2 weeks after daily application of VB-001 for a minimum period of 8 h per day (overnight) compared to a marketed antidandruff shampoo used on alternate days (iii) Change in hair fall, subject satisfaction including hair sensorial evaluation measured by scalp related quality of life index on days 0, 5, 9 and 14. (iv) High resolution photographic monitoring of worst affected area on days 0, 5, 9 and 14 (v) In-use skin safety and tolerability in patients exposed to VB-001 at day 5, 9 and 14 (vi) The incidence of adverse events.

### Patients and study design

This study was a randomized, parallel group, active controlled trial. Protocol approval was obtained from an independent ethics committee established following the guidelines of Helsinki Declaration (1975) before the initiation of the clinical trial (details on Clinical Trial Registry India: CTRI/2013/01/003283). Informed consent from adults and informed assent from children aged ≥ 15 years were likewise secured prior to treatment.

Healthy males or females aged ≥ 15 years and ≤ 65 with a clinical diagnosis of moderate adherent dandruff of the scalp were recruited for the study to monitor the effects of topical antidandruff leave-on formulation VB-001 versus those of marketed antidandruff ZPTO shampoo on moderate adherent dandruff of the scalp at various dermatology clinics in Mumbai and Nashik, India.

Both newly diagnosed and OPD follow-up patients of moderate adherent dandruff of the scalp were screened for enrollment following stated inclusion criteria. Before the initiation of the study, participating doctors and other evaluators were made completely aligned on the scoring matrix to reduce interpretational variability. Six zones on each patient scalp were pre-defined for assessment (right frontal, left frontal, right parietal/temporal, left parietal/temporal, right occipital, left occipital). Comb was used to part the hair in each area to give a clear view of the patient scalp. Each section of the scalp was assessed for the presence of dandruff flakes that were adhering to the scalp skin using a 0 to 5 scale (0 means no flakes and 5 means intense flaking) as described in Table 1. Loose flakes in the hair were not considered in the grading. The final or total ASFS was determined by adding the grades for all six zones on the scalp. Thus, based on the stated grading system, patients with a minimum total ASFS of 6 and not exceeding 14; baseline scaling score of at least 3 and not more than 4 of adherent flaking in at least one zone of the scalp (6 zones- two frontal, two parietal plus temporal and two occipital) were diagnosed with moderate adherent dandruff of the scalp and considered eligible for inclusion. IGA for pruritus score of at least 1 was set as another inclusion criterion. Inclusion screening ensured that the patients had not used an antidandruff agent in the 14 days preceding the trial and they were willing to refrain from use of all other topical medications that would affect the results of the trial, including medicated shampoos/oils or antibiotics, during the treatment and observation periods (from day 0 to day 14).

**Table 1:** ASFS grading scale applicable to the study

Grade	Standard Established
0	No scales
1	Thin scales
2	Diffused thin scales
3	Thick heaped-up scales but not forming plaques
4	Diffused thick heaped-up scales but not forming plaques
5	Very thick heaped-up scales forming plaques

Exclusion criteria precluded the participation of: patients who had a history or presence of Parkinson’s disease, HIV, infections or disorders of the central nervous system, history of overt bacterial, viral or fungal infections of the head/neck, a history or presence of compromising dermatosis elsewhere on the skin; patients with actinically damaged skin; patients with any skin condition that would interfere with the diagnosis or assessment of adherent dandruff; e.g., psoriasis, acne, atopic dermatitis; patients with clinically significant systemic disease (e.g., immunological deficiencies, AIDS, current malignancies, uncontrolled diabetes mellitus); patients who had used within 1 month prior to baseline any treatment that would affect the results of the trial, including 1) systemic antifungal, 2) systemic steroids 3) systemic antibiotics 4) systemic anti-inflammatory agents or 5) cytostatic or immune-modulating drugs (e.g. cyclosporine, tacrolimus, pimecrolimus) 6) topical steroids 7) topical retinoids 8) topical anti-inflammatory agents 9) topical antibiotics or 10) topical treatment of adherent dandruff (e.g., coal tar preparations, antidandruff shampoos/oils/gels/creams/conditioners 11) antihistamines; and patients with any other major medical problem that the investigator deemed likely to increase the risk for adverse events associated with the intervention and pregnant or lactating women by history.

### Materials

VB-001 antidandruff leave-on formulation was developed at Vyome Biosciences Pvt. Ltd by including an ester derivative of a medium chain fatty acid (considered as a GRAS excipient) to a mixture of piroctone olamine (POL) dissolved in absolute alcohol, followed by the addition of a well-accepted topical formulation base to obtain a clear, transparent formulation, free from undissolved solid material. The comparator antidandruff ZPTO shampoo was obtained from marketed batch of a reputed brand. VB-001 was packaged into uniform transparent plastic bottles with a small opening and antidandruff shampoo was repacked in white plastic bottles with a small opening. There were clear differences between VB-001 and antidandruff shampoo in appearance and viscosity.

To harmonize the study and for patient compliance, two more non-antidandruff products from reputed brands were included in both the study arms; a non-antidandruff shampoo for VB-001 arm and a non-antidandruff conditioner for antidandruff shampoo arm. Both non antidandruff products were repacked in similar white plastic bottles as the ones used for antidandruff shampoo. Although the study was an open label, repacking was done just to avoid cognitive bias.

### Randomization, treatment allocation and blinding

**Fig. 1:**
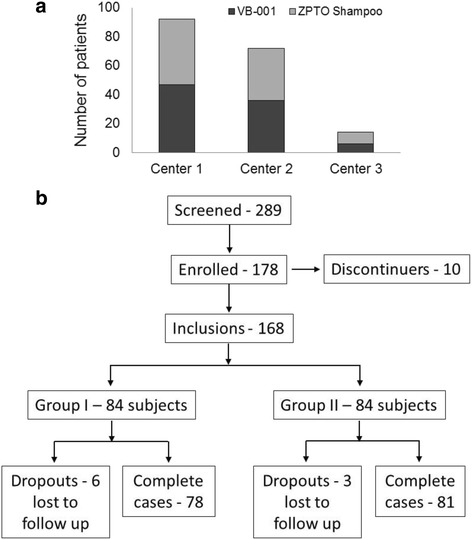
Study population. **a** Graphical representation of the patient randomization at the three centers where the trial was conducted. **b** Trial profile showing subject population. Groups I and II represent VB-001 and ZPTO antidandruff shampoo groups respectively

The patients were recruited in the ratio of 1:1 between the arms. A randomization sheet was generated manually based on ASFS. Patients were allocated to one of the two groups to receive either test product (VB-001) or the comparator product (ZPTO shampoo). The final group wise distribution of the patients recruited at the three centers is depicted in Fig. 1a. The codes were not disclosed to the investigator until the end of study.

### Study Intervention

Each subject was given one bottle (250 ml) of VB-001 or marketed antidandruff shampoo at each visit. For the VB-001 group, the patients were instructed to apply 10 ml of investigational product daily with massage onto the scalp and leave overnight (at least for 8 h leave-on). These patients were further instructed to wash their head on the next day of investigational product application with marketed non-antidandruff shampoo given to them. The patients assigned to the marketed antidandruff shampoo group were instructed to apply sufficient quantity with gentle massage onto scalp, lather and rinse off thoroughly with water on alternate days. These patients were further instructed to gently massage the given non-antidandruff conditioner onto the shampoo cleaned wet hair and rinse off thoroughly with water. Patients in both groups were advised not to apply any other product with antidandruff effect during the study period. All subjects followed their respective procedures for 2 weeks.

### Clinical assessment

Subjects were evaluated at baseline and on day 5, 9 and 14 by the principle investigator. The primary end points of the study were the assessment of clinical efficacy of the investigational product and marketed antidandruff shampoo in terms of demonstrated improvement in dandruff score and pruritus in both groups. Secondary end points included efficacy and safety of investigational product from baseline in terms of changes in hair sensorial, hair fall and the incidence of adverse events during the study in both groups. The study end points were recorded and digital photographs of affected areas of the scalp were obtained at every visit in both groups.

The clinical severity of dandruff was measured using mean ASFS on scalp using a scale (0: No score- 5: Very thick heaped up scales forming plaques). The scores on 6 zones (right frontal, left frontal, right parietal/temporal, left parietal/temporal, right occipital, left occipital) of scalp were individually measured and total score recorded at baseline. Any further improvement was measured at day 5, 9 and 14. Clinical efficacy of pruritus was measured using IGA for pruritus and subjects with IGA for pruritus score of at least 1 were considered at baseline. Further improvement was measured at 5, 9 and 14 days.

A scalp related quality of life questionnaire was circulated among subjects in order to capture their perception on change in hair fall, overall satisfaction and hair sensorial on day 0, 5, 9 and 14. The Digital Viewer III 2.0 M digital magnascope is a simple microscope and was used to obtain magnified images of hair and scalp. High resolution photographic monitoring of worst affected area was performed on days 0, 5, 9 and 14 using the digital magnascope. The in-use skin safety and tolerability in subjects exposed to VB-001 as adverse events or skin intolerances were reported during the study period. The efficacy and safety assessments used in this study were standard, i.e., widely used and generally recognized as reliable, accurate, and relevant (able to discriminate between effective and ineffective agents).

### Stopping guidelines

No stopping guidelines were envisaged for this study.

Eligible subjects, who for whatever reason, did not report to the clinic to receive the first application of test product and comparator product after having been selected for the study were referred to as discontinuers in the report. A volunteer who was withdrawn from the study, for whatever reason, was classified as a drop out, and identified as such in the relevant Case Report Form (CRF).

### Sample size

The sample size was calculated accepting a power of 80%, with an alpha of 0.05, using the formula for computing the difference between two proportions the aim was to recruit 84 subjects per arm to allow for a 10% drop out rate.

### Data processing and analysis

Data from the study were subjected to both per protocol (PP) analysis and intention to treat (ITT) analysis and results were found to be comparable. Further interpretation of data was drawn based on PP analysis wherein all ASFS and pruritus scores for each patient on each visit was normalized to their individual baseline (day 0) score and the normalized data sets were further analyzed for differences. Tests of significance for difference between the two groups were performed using Student’s *t*-test while analyzing the normalized ASFS and pruritus data. Analysis of the data from the quality of life questionnaire were performed using ANOVA Kruskal Wallis test. Further, the data for monitoring hair fall and hair softness was analyzed using Chi square test as they represented categorical variables.

## Results

### Study population

Of the 289 individuals who were screened, 178 met the entry criteria and were eligible subjects but 10 subjects, for whatever reason, did not report to the clinic to receive the first application of test product or comparator product after having been selected for the study were referred to as discontinuers in the report and 168 subjects were randomized to the treatment (VB-001, *n* = 84) and control (marketed antidandruff shampoo, *n* = 84) groups (Fig. 1b). Of these, 9 were lost to follow-up and were considered drop outs. A PP analysis was performed in which 159 subjects were included. No statistical difference was observed in the baseline demographics of the study population as summarized in Table 2.

**Table 2:** Demographic characteristic of the study population (*n* = 168) at baseline

Parameters		VB-001 Group	ZPTO Shampoo Group	*p* value (Between groups)
No. of cases		84	84	
Age (Yrs)	Mean SD Range	26.20 09.37 15–58 yrs	25.31 07.37 15–46 yrs	-
Gender (%)	Male Female	10 (11.9) 74 (88.1)	14 (16.7) 70 (83.3)	-
Mean total ASFS (Mean ± SD)		11.09 ± 1.55	10.86 ± 1.53	0.3479 (NS)
Mean score for pruritus (Mean ± SD)		1.19 ± 0.40	1.17 ± 0.38	0.7471 (NS)
Mean scalp related quality of life index score-II (Mean ± SD)		12.05 ± 3.18	11.67 ± 3.13	0.4489 (NS)

*NS* non significant by ANOVA Kruskal Wallis test

### Clinical effects

#### VB-001 imparted consistently better reduction in ASFS

**Fig. 2:**
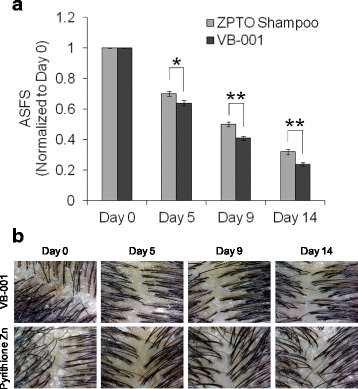
Clinical efficacy of VB-001 in seborrheic dermatitis of the scalp. **a** Average Adherent Scaling and Flaking Score (ASFS) of patients in ZPTO shampoo group (*gray bars*; *n* = 81) and patients in VB-001 group (*black bars*; *n* = 78). Values represent group average ± SEM of individual ASFS on each day normalized to their individual score on day 0. Statistical analysis was performed using Student’s *t*-test [* *p* < 0.05; ** *p* < 0.001]. **b** High resolution scalp images of patients treated with VB-001 (*top panel*) or antidandruff shampoo containing zinc pyrithione (*lower panel*) captured using a digital magnascope on the indicated days of the clinical trial

Mean total ASFS decreased from baseline in both VB-001 and marketed antidandruff ZPTO shampoo groups (Fig. 2a). However, the percentage reductions from baseline in the VB-001 group were significantly higher at all measurement time-points. The post-treatment reduction in the mean ASFS value from baseline in the VB-001 group was 76%, which was significantly higher (*P* < 0.001) than that observed in the marketed antidandruff ZPTO shampoo group (68%) as depicted in Fig. 2a. High-resolution photographic monitoring of worst affected scalp area revealed a dramatic reduction in adherent flaking over time following treatment in both groups (Fig. 2b).

**Fig. 3:**
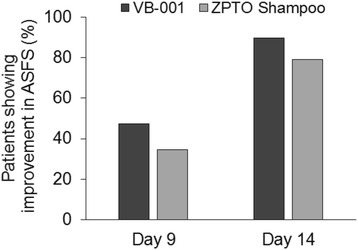
Comparison of patients showing improvement in scalp flaking during the course of the study. Bar graphs show percentage of patients reaching an ASFS of less than 5 at the indicated days of the trial in VB-001 group versus ZPTO shampoo group

In addition, when the efficacy of each product was assessed by comparing the proportion of subjects who achieved reduction in ASFS (score ˂ 5) it was found that 47% of the patients (37 out of 78) in VB-001 group and 34% (28 out of 81) in marketed antidandruff ZPTO shampoo group showed an ASFS of less than 5 on day 9. In VB-001 group 90% subjects and 79% in marketed antidandruff ZPTO shampoo group showed a good response (ASFS < 5) at day 14 (Fig. 3 and Table 3).

**Table 3:** Proportion of subjects showing reduction in flaking and itching in both groups

Day	VB-001 (%)	ZPTO shampoo (%)
	ASFS ˂ 5	
Day 5	7	6
Day 9	47	34
Day 14	90	79
	Pruritus IGA Score = 0	
Day 5	27	10
Day 9	68	53
Day 14	87	85

#### VB-001 treatment enabled an early reduction in pruritus

**Fig. 4:**
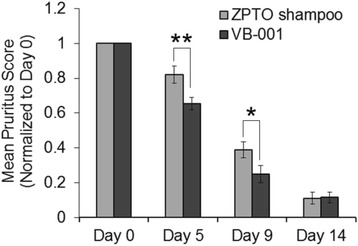
Clinical efficacy of VB-001 in relief of pruritus in dandruff afflicted scalp. Mean pruritus score of patients in ZPTO shampoo group (*gray bars*) and patients in VB-001 group (*black bars*). Values represent group average ± SEM of individual pruritus score on each day normalized to their individual score on day 0. Statistical analysis was performed using Student’s *t*-test [* *p* < 0.05; ** *p* < 0.01]

Pruritus symptom was analyzed by score of pruritus- Investigator global assessment (IGA). Mean score for pruritus (normalized to baseline) decreased from baseline to day 14 in both groups (Fig. 4). The percentage reductions from baseline in the VB-001 group were significantly higher at day 5 and day 9 time-points. Mean score for pruritus showed a significant fall of 35% among VB-001 group and 18% in ZPTO shampoo group from baseline after 5 days of treatment which was significantly more for VB-001 group than the shampoo group (*P* < 0.01). Similarly, after 9 days of treatment, mean score for pruritus showed a significant fall from baseline in both the groups i.e. 75% among VB-001 group and 61% in ZPTO shampoo group which was significantly more (*P* < 0.05) in VB-001 group than ZPTO shampoo group (Fig. 4). However, at the end of day 14, mean score for pruritus showed a significant fall of 89.1% and 87.2% from baseline among VB-001 group and ZPTO shampoo group respectively. There was no significant difference between VB-001 group and ZPTO shampoo group at day 14.

**Fig. 5:**
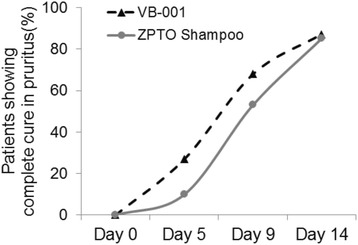
Comparison of patients achieving complete cure in pruritus during the course of the study. Graphs show percentage of patients reaching an IGA pruritus score of zero at the indicated days of the trial in VB-001 group versus ZPTO shampoo group

In addition, the efficacy of each product was assessed by comparing the proportion of subjects who achieved reduction in itching (score = 0) which was remarkable at day 5 and day 9 in the VB001 group. 26.9% of the subjects in VB-001 showed complete cure in pruritus on day 5 compared to only 9.9% in the ZPTO shampoo group (Fig. 5 and Table 3). Similarly, on day 9, a greater percentage of subjects (67.9%) in the VB-001 group showed complete cure in pruritus compared to the 53.1% in ZPTO shampoo group. On day 14 both VB-001 group (87.2% subjects) and marketed antidandruff shampoo group (85.2% subjects) showed an excellent response (Fig. 5).

#### VB-001 improved patient experience during antidandruff therapy

To establish subject satisfaction in both groups the sensorial effects and hair fall change was measured by Scalp Related Quality Of Life Index. The result for the mean total score of scalp related quality of life index showed that there was a significant improvement in mean total score of Scalp Related Quality of Life Index in both groups as compared to baseline at all time points (day 5, day 9 and day 14). At day 5 and day 9, the results were comparable between both groups. After 14 days of treatment, however, mean scalp related quality of life index score-II showed a significant increase of 51.6% and 45.1% from baseline in VB-001 group and ZPTO shampoo group, respectively (Table 4). The rise was significantly more in VB-001 group than ZPTO shampoo group. Additionally, 14 days post treatment, 41% of subjects in VB-001 group reported a visible reduction in hair fall which was significantly more than the 18.5% subjects in the antidandruff shampoo group. Finally, 83.3% of subjects in VB-001 group reported softness of hair which was significantly more than the 67.9% subjects in the ZPTO shampoo arm (Table 4).

**Table 4:** Treatment success in both groups

Efficacy Parameter	VB-001 (*N* = 78)	ZPTO shampoo (*N* = 81)	*P* value
Mean ASFS (normalized) difference (Baseline-Day 14)	75.6%	67.9%	0.0005^a^
Mean pruritus score (normalized) difference (Baseline-Day 9)	75%	61%	0.03^a^
Mean scalp related quality of life index score difference (Baseline-Day 14)	51.6%	45.1%	0.03^b^
Visible hair fall reduction at day 14 (% of subjects)	41%	18.5%	<0.05^c^
Softness at day 14 (% of subjects)	83.3%	67.9%	<0.05^c^

^a^Significant by *t*-test; ^b^Significant by ANOVA Kruskal Wallis; ^c^Significant by Chi square test

### Safety

There were no adverse events or skin intolerances reported during the study period. Both product regimens were well tolerated by the subjects.

## Discussion

The accepted scientific consensus is that *Malassezia* is the causative agent for dandruff and thus for multiple decades the preferred modes of treatment have included the use of anti-fungal agents [7, 8], many of which show good efficacy. However, the current understanding is that dandruff occurs in susceptible individuals who have decreased scalp lipid levels and inherent defects in stratum corneum (SC) permeability barrier [4, 5]. Such defects in skin allow pathogenic colonization by the otherwise commensal *Malassezia* spp. which exacerbates the damage in an already impaired SC barrier to present features of dandruff. Recovery of skin from dandruff-associated symptoms by use of topical anti-fungal agents is primarily due to removal of *Malassezia* and the resultant secondary effects from clearing of the microbe [7]. However, recurrence of dandruff is common in individuals treated solely with an anti-fungal agent possibly due to (apart from other reasons) persistent SC defects that are not addressed by the anti-fungal treatment.

Multiple evidences, as reviewed by many [7, 8], suggest that the use of synthetic anti-fungal agents efficiently works against the microbe but commonly used vehicle formulation(s) alter the lipid balance in the affected SC and subsequent trans-epidermal water loss (TEWL) issue remains unaddressed [4]. Harsh treatment from surfactants in traditional shampoo formulations further disturbs the free lipid levels on an already damaged SC [11, 12]. Such deteriorated scalp condition also instigates hyper-proliferation of keratinocytes, changes the maturation pattern of corneocytes and subsequently leads to the manifestation of inflammation [13]. Additionally, many currently available anti-dandruff treatment options show limited efficacy due to factors like short contact time (wash-off formulations), rising fungal resistance to various anti-fungals, formulation challenges etc. Hence there is clear scope to improve anti-dandruff treatment options using formulations that target the pathogen as well as the host predisposition.

VB-001 is a well-researched topical formulation designed by keeping in mind the recent literature about *Malassezia* genetics/physiology [6] and the evolving understanding about the benefits of a leave-on product in the treatment of dandruff. The clinical study results presented herein indicate that although both treatments showed beneficial effects on ASFS, pruritus and scalp related quality of life index score, VB-001 was significantly better in improving all outcomes compared to a leading ZPTO anti-dandruff shampoo formulation. The most apparent outcome was the early onset of relief of symptoms in the VB-001-treated group compared to the ZPTO shampoo-treated group. The effect of VB-001 started as early as day 5 for relief of pruritus symptoms. The faster onset of action is possibly attributable to the longer contact time of the antifungal agent in VB-001 (leave-on formulation). An early onset of the relief of symptoms is much coveted by patients and often dictates compliance to a certain therapeutic regimen. Such data encourages further development of similar formulations to achieve overall better efficacy & patient satisfaction. On the contrary, shampoo formulations are known to have a very short resident time on the scalp and additionally require a rinsing step that makes achievement of effective concentrations of actives a challenging task.

Additionally, VB-001 includes a potent anti-*Malassezia* medium chain fatty acid derivative as a GRAS (generally regarded as safe) excipient and demonstrated enhanced in vitro fungal killing activity when compared to a similar formulation containing the active (piroctone olamine) alone (data not shown). The overall extra efficacy of VB-001 reported in this study may be ascribed, in part, to the presence of this excipient. Finally, VB-001 is unique in its composition by being devoid of long chain fatty acids (LCFAs) or their derivatives. This feature ensures that the formulation does not provide any nutritional benefits to *Malassezia* residing on the scalp as LCFAs are known to promote growth of this yeast [9]. Hence modification of the scalp microenvironment through molecular replacement of sebaceous fatty acids with the anti-*Malassezia* fatty acid derivative may enable efficient clearance of the fungus and thus translate into the observed therapeutic benefits.

With respect to host parameters, although there are reports which suggest that the new generation of ZPTO shampoo takes care of many of the barrier defect issues [14, 15] but it seems to be an indirect effect following elimination of the causal pathogen from the scalp. On the other hand, a leave-on formulation with proper balance of all or some of the three main classes of moisturizing agents (occlusives, humectants and emollients) would leave a layer on the scalp which would help slow down the movement of water in and out of the scalp [12, 13] and enhance the patient experience. Oil based formulations have been earlier reported to impart properties of occlusives, coat the SC and retard TEWL [16]. Recent reports of skin cleansing agents also suggest that the addition of saturated fatty acids like palmitic or stearic acid can replenish the lost lipid from the scalp and rejuvenate skin health [17]. But the addition of LCFAs to antidandruff formulations may inadvertently provide nutritional advantages to the pathogen that the treatment is intended to control. VB-001, on the other hand, is devoid of any long chain fatty acid and yet it performed better than the marketed ZPTO shampoo formulation in all scalp related quality of life parameters evaluated in the study. Therefore, VB-001, a non-greasy, oil-based leave-on formulation with carefully chosen ingredients may provide unique advantages over the existing best in class therapy by not only depriving *Malasezzia* from its coveted nutrients but also addressing the pre-existing issue of loss in skin hydration.

Although randomization of the trial was done manually, both the treatment groups were more or less matched in terms of baseline demographics. However, there were big differences in the rate of recruitment of male and female patients in the study. In order to minimize any discrepancy in the outcome, the ratio of male to female was maintained in a similar range for both groups (Table 2).

The open label feature of the study was a limitation of the trial. However, the study design involved the testing of VB-001 against a comparator product (best in class), wherein the comparator was used as per published protocol that produces maximal efficacy. Additionally, this was not only a formulation versus formulation study but also a regimen versus regimen study. It was assumed that the marketed comparator had optimized their frequency of use in the wash-off format to achieve desirable results. The goal was to test whether the new leave-on product is as good as or better than the existing wash-off shampoo product. This makes the study design rigorous despite the open label.

## Conclusion

VB-001 is, thus, a novel, clinically proven effective and safe formulation, and has the potential to emerge as an attractive novel treatment for moderate adherent dandruff.

## Abbreviations

ASFS: Adherent scalp flaking score
CTRI: Clinical trials registry -India
GRAS: Generally regarded as safe
IGA: Investigators’ global assessment
ITT: Intention to treat
LCFA: Long chain fatty acid
OPD: Out-patient department
PP: Per protocol
SC: stratum corneum
TEWL: Trans-epidermal water loss
ZPTO: Zinc pyrithione

## References

[1] Piérard-Franchimont C, Xhauflaire-Uhoda E, Piérard GE (2006). Revisiting dandruff. Int J Cosmet Sci.

[2] McGinley KJ, Leyden JJ, Marples RR, Kligman AM (1975). Quantitative microbiology of the scalp in non-dandruff, dandruff, and seborrheic dermatitis. J Invest Dermatol.

[3] Ro BI, Dawson TL (2005). The role of sebaceous gland activity and scalp microfloral metabolism in the etiology of seborrheic dermatitis and dandruff. J Investig Dermatol Symp Proc.

[4] Harding CR, Moore AE, Rogers JS, Meldrum H, Scott AE, McGlone FP (2002). Dandruff: a condition characterized by decreased levels of intercellular lipids in scalp stratum corneum and impaired barrier function. Arch Dermatol Res.

[5] DeAngelis YM, Gemmer CM, Kaczvinsky JR, Kenneally DC, Schwartz JR, Dawson TL (2005). Three etiologic facets of dandruff and seborrheic dermatitis: Malassezia fungi, sebaceous lipids, and individual sensitivity. J Investig Dermatol Symp Proc.

[6] Xu J, Saunders CW, Hu P, Grant RA, Boekhout T, Kuramae EE (2007). Dandruff-associated Malassezia genomes reveal convergent and divergent virulence traits shared with plant and human fungal pathogens. Proc Natl Acad Sci U S A.

[7] Shuster S (1984). The aetiology of dandruff and the mode of action of therapeutic agents. Br J Dermatol.

[8] Ranganathan S, Mukhopadhyay T (2010). Dandruff: the most commercially exploited skin disease. Indian J Dermatol.

[9] Papavassilis C, Mach KK, Mayser PA (1999). Medium-chain triglycerides inhibit growth of Malassezia: implications for prevention of systemic infection. Crit Care Med.

[10] Mayser P (2015). Medium chain fatty acid ethyl esters - activation of antimicrobial effects by Malassezia enzymes. Mycoses.

[11] Misra M, Ananthapadmanabhan KP, Hoyberg K, Gursky RP, Prowell S, Aronson M (1997). Correlation between surfactant-induced ultrastructural changes in epidermis and transepidermal water loss. J Soc Cosmet Chem.

[12] Del Rosso JQ, Levin J (2011). The clinical relevance of maintaining the functional integrity of the stratum corneum in both healthy and disease-affected skin. J Clin Aesthet Dermatol.

[13] Turner GA, Hoptroff M, Harding CR (2012). Stratum corneum dysfunction in dandruff. Int J Cosmet Sci.

[14] Warner RR, Schwartz JR, Boissy Y, Dawson TL (2001). Dandruff has an altered stratum corneum ultrastructure that is improved with zinc pyrithione shampoo. J Am Acad Dermatol.

[15] Billhimer W, Erb J, Bacon R (2006). Shampooing with pyrithione zinc reduces trans-epidermal water loss in scalp of dandruff-involved patients. J Am Acad Dermatol.

[16] Schliemann-Willers S, Wigger-Alberti W, Kleesz P, Grieshaber R, Elsner P (2002). Natural vegetable fats in the prevention of irritant contact dermatitis. Contact Dermatitis.

[17] Mukherjee S, Edmunds M, Lei X, Ottaviani MF, Ananthapadmanabhan KP, Turro NJ (2010). Stearic acid delivery to corneum from a mild and moisturizing cleanser. J Cosmet Dermatol.

